# Elevated *APOBEC3B* Correlates with Poor Outcomes for Estrogen-Receptor-Positive Breast Cancers

**DOI:** 10.1007/s12672-014-0196-8

**Published:** 2014-08-15

**Authors:** Anieta M. Sieuwerts, Scooter Willis, Michael B. Burns, Maxime P. Look, Marion E. Meijer-Van Gelder, Andreas Schlicker, Marinus R. Heideman, Heinz Jacobs, Lodewyk Wessels, Brian Leyland-Jones, Kathryn P. Gray, John A. Foekens, Reuben S. Harris, John W. M. Martens

**Affiliations:** 1 Department of Medical Oncology and Cancer Genomics Netherlands, Erasmus MC Cancer Institute, Dr. Molewaterplein 50, 3015 GE Rotterdam, Netherlands; 2 Department of Biochemistry, Molecular Biology, and Biophysics, Masonic Cancer Center, Institute for Molecular Virology, University of Minnesota, Minneapolis, MN 55455 USA; 3 The Netherlands Cancer Institute, 1066 CX Amsterdam, Netherlands; 4 Department of Molecular and Experimental Medicine, Avera Cancer Institute, Sioux Falls, SD 57105 USA; 5 International Breast Cancer Study Group (IBCSG) Statistical Center, Department of Biostatistics and Computational Biology, Dana-Farber Cancer Institute, Harvard School of Public Health, Boston, MA 02215 USA

**Keywords:** Breast Cancer, Overall Survival, Adjuvant Systemic Therapy, Copy Number Aberration, HNPCC Tumor

## Abstract

Recent observations connected DNA cytosine deaminase APOBEC3B to the genetic evolution of breast cancer. We addressed whether *APOBEC3B* is associated with breast cancer clinical outcomes. *APOBEC3B* messenger RNA (mRNA) levels were related in 1,491 primary breast cancers to disease-free (DFS), metastasis-free (MFS), and overall survival (OS). For independent validation, *APOBEC3B* mRNA expression was associated with patient outcome data in five additional cohorts (over 3,500 breast cancer cases). In univariate Cox regression analysis, increasing *APOBEC3B* expression as a continuous variable was associated with worse DFS, MFS, and OS (hazard ratio [HR] = 1.20, 1.21, and 1.24, respectively; all *P* < .001). Also, in untreated ER-positive (ER+), but not in ER−, lymph-node-negative patients, high *APOBEC3B* levels were associated with a poor DFS (continuous variable: HR = 1.29, *P* = .001; dichotomized at the median level, HR = 1.66, *P* = .0002). This implies that *APOBEC3B* is a marker of pure prognosis in ER + disease. These findings were confirmed in the analyses of five independent patient sets. In these analyses, *APOBEC3B* expression dichotomized at the median level was associated with adverse outcomes (METABRIC discovery and validation, 788 and 706 ER + cases, disease-specific survival (DSS), HR = 1.77 and HR = 1.77, respectively, both *P* < .001; Affymetrix dataset, 754 ER + cases, DFS, HR = 1.57, *P* = 2.46E-04; NKI295, 181 ER + cases, DFS, HR = 1.72, *P* = .054; and BIG 1-98, 1,219 ER + cases, breast-cancer-free interval (BCFI), HR = 1.42, *P* = 0.0079). *APOBEC3B* is a marker of pure prognosis and poor outcomes for ER + breast cancer, which strongly suggests that genetic aberrations induced by *APOBEC3B* contribute to breast cancer progression.

**Electronic supplementary material:**

The online version of this article (doi: 10.1007/s12672-014-0196-8) contains supplementary material, which is available to authorized users.

## Introduction

Clinical heterogeneity is a confounding hallmark of breast cancer. This variation in disease manifestation, also true for many other cancers, is mirrored in the cancer genome with hundreds to thousands of somatic mutations in each tumor. The mutations involved are mostly base substitutions, but also include small insertions and deletions, larger-scale events such as translocations, and catastrophic events such as chromothripsis and kataegis [34, 4]. Several recent studies identified the APOBEC deaminase family as a major enzymatic source of somatic driver and passenger mutations in breast cancer. First, Sanger sequencing studies indicated a cytosine-biased mutation pattern dominated by C-to-T transition mutations [12, 33]. Second, next-generation full genome sequencing studies revealed strand-coordinated cytosine mutation clusters (called kataegis), consisting predominantly of C-to-T transitions and C-to-G transversions within 5′-TC dinucleotide motifs [20]. Third, the antiviral DNA cytosine deaminase apolipoprotein B messenger RNA (mRNA) editing enzyme catalytic polypeptide-like 3B (APOBEC3B) was shown to be overexpressed in cell lines and primary breast tumors and responsible for elevated levels of genomic uracil and mutations in cell lines. This correlated with increased mutational loads in primary tumors [2]. Fourth, APOBEC3B overexpression caused increased mutational loads, cell cycle deviations, induction of DNA damage markers, and ultimately cell death [2, 36, 30]. Finally, recent sequencing meta-analyses data have underscored the importance of APOBEC3B in causing both the dispersed and clustered mutations in breast cancer and also implicated it as a dominant mutagen in several additional cancers [3, 28, 1].

APOBEC3B is a member of a larger family of polynucleotide cytosine deaminases with diverse physiological functions in innate and adaptive immunity, lipid metabolism, and heart development [24, 7]. The APOBEC3 subfamily consists of seven members, APOBEC3A, APOBEC3B, APOBEC3C, APOBEC3D, APOBEC3F, APOBEC3G, and APOBEC3H [6, 16]. APOBEC family members are generally thought of as innate immune effectors with demonstrated single-stranded DNA cytosine to uracil (C-to-U) editing activity and the capacity to restrict the replication of a diverse array of transposons and viruses [11, 35]. APOBEC2 has not yet been demonstrated to elicit biochemical activity, but the mouse knockout suggests function in cardiovascular muscle development [38, 10, 29]. The family namesake, APOBEC1, is capable of editing both DNA and RNA cytosines, with a general role in innate immunity and a specialized role in *APOB* mRNA editing [24]. Finally, a last member of the APOBEC protein family, AID (activation-induced deaminase), is a DNA cytosine deaminase that targets rearranged immunoglobulin gene variables and switches region sequences to mediate the distinct processes of somatic hypermutation and class switch recombination, which are central to antibody affinity maturation and effector functions, respectively [9, 27].

As a potential continuous source of genetic aberrations in breast cancer, we hypothesized that APOBEC3B overexpression may accelerate cancer progression and lead to poor clinical outcomes. To test this hypothesis, we quantified *APOBEC3B* mRNA levels using reverse-transcriptase-quantitative PCR (RT-qPCR) in a large series of primary breast tumors and asked whether expression levels correlate with disease outcome. To probe the potential link between *APOBEC3B* mRNA levels and pure disease prognosis, i.e., to study the relation with the natural course of the disease, primary tumors of lymph-node-negative (LNN) breast cancer patients who did not receive systemic adjuvant therapy were evaluated separately. To provide independent validation, we analyzed five additional cohorts representing three distinct platforms (Illumina, Affymetrix, Agilent) for *APOBEC3B* mRNA expression measured by gene-specific probes and their association with patient outcome data. The combined results indicate that high levels of *APOBEC3B* mRNA expression are a significant prognostic biomarker of poor breast cancer outcomes, exclusively in cases with ER-positive primary disease.

## Patients and Methods

### RT-qPCR Cohort (or Rotterdam Cohort)

One thousand four hundred ninety-one tumor specimens obtained at primary surgery between 1978 and 2000 were selected from our liquid nitrogen tumor bank at the Erasmus MC (Rotterdam, Netherlands). Inclusion criteria were invasive breast cancer with freshly frozen tissue available irrespective of nodal status, tumor size, and type of adjuvant systemic therapy. Major details of this cohort have been described before [32], and patient and patho-clinical characteristics are presented in Table 1. Our institution’s Medical Ethical Committee approved our protocol for studying molecular markers associated with disease recurrence in anonymized tumor tissues (MEC 02 · 953). In this study, we adhere to the Code of Conduct of the Federation of Medical Scientific Societies in the Netherlands (http://www.fmwv.nl/) and report in accordance with the REMARK criteria on clinical reporting [19] (see Supplementary file and Diagram S1).

**Table 1:** Patient characteristics and their relationship with *APOBEC3B* mRNA expression

Characteristics	No. of patients (%)	Median levels (interquartile range)	*P* value
All patients	1,491 (100)	0.22 (0.27)	
Age (years)			NS^a^
≤40	190 (12.7)	0.26 (0.36)	
41–55	558 (37.3)	0.21 (0.26)	
56–70	492 (33.0)	0.21 (0.29)	
≥71	251 (16.8)	0.20 (0.23)	
Menopausal status			NS^b^
Premenopausal	632 (42.4)	0.22 (0.29)	
Postmenopausal	859 (57.6)	0.21 (0.26)	
Nodal status			.009^b^
N 0	829 (55.6)	0.20 (0.26)	
N 1–3	298 (20.0)	0.22 (0.26)	
N >3	364 (22.4)	0.25 (0.28)	
Tumor size			<.001^b^
pT1	512 (34.3)	0.19 (0.24)	
pT2 + unknown	821 (55.0)	0.24 (0.30)	
pT3/pT4	158 (10.6)	0.25 (0.30)	
Tumor grade^c^			<.001^b^
Good/moderate	232 (15.5)	0.18 (0.22)	
Unknown	450 (30.1)	0.21 (0.24)	
Poor	809 (54.2)	0.24 (0.32)	
Estrogen receptor status^d^ (*r*_s_ = −0.31)^a^			< .001^a^
ER negative	324 (21.7)	0.41 (0.60)	
ER positive	1167 (78.3)	0.19 (0.21)	
Progesterone receptor status^d^ (*r*_s_ = −0.45)^a^			<.001^a^
PR negative	554 (37.2)	0.35 (0.46)	
PR positive	937 (62.8)	0.16 (0.18)	

*NS* not significant

^a^Spearman rank correlation test

^b^Two-sample Wilcoxon rank-sum (Mann-Whitney) test followed by a test for trend if appropriate

^c^Good/moderate tumor grade was compared to poor grade

^d^ER and PR cutoff based on mRNA expression as described [32]

### RNA Extraction, cDNA Synthesis, and Quantification by RT-qPCR

The detailed procedure for tissue processing, RNA extraction, cDNA synthesis and quantification of *APOBEC3B* mRNA transcripts by RT-qPCR has been described [31] (see supplement for detailed description).

### Validation Cohorts

To validate our findings, we used five published datasets that contained both microarray and clinical follow-up data. In these data sets, different primary endpoints were reported, and various systemic adjuvant therapies were applied [8, 37, 13, 26].

The Illumina HT-12 v4 microarray data sets reported by Curtis et al. [8], consisting of two independent data sets, called METABRIC discovery (*n* = 997) and METABRIC validation (*n* = 995), were used. The METABRIC clinical data includes immunohistochemistry (IHC) ER status as well as disease-specific (DSS) survival data, where all reported deaths are attributed to breast cancer and deaths from other causes are censored. Here, we analyzed ER + samples to establish a METABRIC discovery ER + sub-cohort (*n* = 788, events = 218) and a METABRIC validation ER + sub-cohort (*n* = 706, events = 214). To determine *APOBEC3B* mRNA expression values, microarray probe expression values were used as published [8].

For the NKI295 microarray cohort, Agilent microarray probe mRNA expression values of *APOBEC3B*, and patient disease-free survival (DFS) of 181 ER + cases according to IHC, were taken from Van de Vijver et al. [37]. Also, here, microarray probe expression values were used as a measure of *APOBEC3B* mRNA expression value. Patients who had died without evidence of disease or who were lost during follow-up were censored at last follow-up in DFS analysis.

The Kaplan-Meier Plotter online service (http://kmplot.com) was used to asses *APOBEC3B* expression as a prognostic biomarker from Affymetrix microarrays manually curated and combined from publically available Gene Expression Omnibus (GEO) cohorts [13]. GEO cohorts overlapping with our Rotterdam cohort mentioned above were excluded. In total, the assembled Affymetrix microarray cohort included in the current study contains 754 ER + samples with DFS as outcome. Microarray probe expression values were used as downloaded from the kmplot web site.

BIG 1-98 was a prospective randomized, phase III, double-blind trial of 8,010 postmenopausal women with hormone-receptor-positive early breast cancer that compares 5 years of adjuvant tamoxifen or letrozole monotherapy or sequential treatment with 2 years of one of these agents followed by 3 years of the other between 1998 and 2003 [26]. Formalin-fixed, paraffin-embedded (FFPE) tissue samples (events = 257) were obtained. Due to overall low recurrence rates in BIG 1-98, a case-cohort sampling was used, where all cases (recurrences) with available RNA materials were included, while non-recurrence cases were sampled according to stratification factors. Included RNA samples were profiled using the Illumina Whole Genome DASL protocol on the Illumina HT-12 v4 microarray. Samples were cubic spline normalized with no background correction using BeadStudio software (Illumina). Twelve hundred nineteen samples were included in the analysis. The association of the expression data with time to disease recurrence (breast-cancer-free interval, BCFI) was assessed using a weighted analysis methods (generalized Horvitz-Thompson methods) to adjust estimates and test statistics to obtain unbiased analyses [28]. The endpoint BCFI was defined as the time from randomization to the first breast cancer event including invasive breast cancer recurrence at local, regional, or distant sites or a new invasive cancer in the contralateral breast, and ignored second (non-breast) malignancies, censored at death without a prior cancer event or last follow-up visit.

### Statistical Analyses

Univariate and multivariate Cox regression analysis was used to assess the association of *APOBEC3B* mRNA expression levels and/or established clinical-pathological factors such as nodal status, age, tumor size, grade, and hormone receptor expression with DFS, metastasis-free survival (MFS), or overall survival (OS). The Cox proportional hazards model was used to calculate the hazard ratios (HRs) and their 95 % confidence intervals (95 % CIs) of covariates in the analyses of DFS, MFS, or OS. Likelihood ratio test was performed to test whether *APOBEC3B* mRNA expression or other covariates were related to the hazard. Survival curves were constructed from DFS, MFS, and OS data using the Kaplan-Meier estimator for survival [14]. Log-rank test was used to test for significant differences between two survival curves. All *P* values are two-sided. For the Rotterdam cohort, the STATA statistical package v.11 was used; for validation, Kaplan-Meier survival analysis BioJava was used which implements the R survival package [23] with the exception for the BIG 1-98 where a weighted analysis as described above was performed.

## Results

### Association of *APOBEC3B* mRNA Expression Levels with Patient and Clinical and Pathological Characteristics

*APOBEC3B* mRNA expression levels were quantified in the Rotterdam cohort (*n* = 1,491) by RT-qPCR of total RNA samples extracted from freshly frozen tumor tissues from patients with primary breast cancer [32]. The primers used here are new but robust because they showed near-identical specificity and efficiency in comparison to previously validated *APOBEC3B* RT-qPCR primers [25, 3] (Supplementary Fig. S1). In this cohort, mRNA expression of *APOBEC3B* was positively correlated with nodal status (*P* = .009), tumor size (*P* < .001), and grade (*P* < .001) and negatively with both ER (*P* < .001) and PR (*P* < .001) (Table 1).

### Association of *APOBEC3B* mRNA Expression Levels with Clinical Outcomes

To determine whether *APOBEC3B* mRNA expression was associated with clinical outcome in breast cancer, we related log-transformed values of *APOBEC3B* using Cox regression analysis with DFS, MFS, and OS. This analysis, using data from all 1,491 patients, showed that higher expression levels of *APOBEC3B* mRNA as a continuous variable were associated with worse DFS, MFS, and OS (HR = 1.20, 95 % CI = 1.11–1.29; HR = 1.21, 95 % CI = 1.11–1.31; and HR = 1.24, 95 % CI = 1.13–1.36; all *P* < .001).

Because a percentage of patients from the Rotterdam cohort received adjuvant treatment, which may confound data analyses, we restricted subsequent analyses to the 829 patients with LNN disease who did not receive any (neo)adjuvant systemic therapy. Of note, this sub-cohort is relatively unbiased because patients with LNN disease at the time this retrospective cohort was collected (1978–2000) did not receive any (neo)adjuvant systemic therapy according to the guidelines in the Netherlands at that time. Thus, analysis of this sub-cohort allowed us to determine the association of *APOBEC3B* expression with the natural course of disease. The median mRNA expression level was used as an unbiased means to split the cohort into *APOBEC3B*-low and *APOBEC3B*-high expression groups. In addition, we stratified the cohort based on ER status because ER + (*n* = 633) and ER − (*n* = 196) breast cancers are biologically distinct diseases [22, 39].

In the 633 LNN patients with ER + disease, *APOBEC3B* mRNA expression split at the median level of the whole cohort was significantly associated with poor DFS (HR = 1.55, 95 % CI = 1.23–1.96, *P* < .001, Table 2), MFS (HR = 1.66, 95 % CI = 1.26–2.17, *P* < .001, Supplementary Table S2), and OS (HR = 1.68, 95 % CI = 1.25–2.24, *P* < .001 (Supplementary Table S3). In 196 LNN cases with ER − breast cancer, no significant association was observed with any of the endpoints. In multivariate Cox regression analysis, together with current prognostic markers such age, tumor size, and grade, steroid hormone receptors and *APOBEC3B* mRNA expression remained significant in ER + breast cancer in the analysis of DFS (HR = 1.32, 95 % CI = 1.02–1.69, *P* = .034, Table 2), MFS (HR = 1.43, 95 % CI = 1.07–1.91, *P* = .015, Supplementary Table S2), and OS (HR = 1.44, 95 % CI = 1.06–1.96, *P* = .02, Supplementary Table S3).

**Table 2:** Univariate and multivariate analysis for disease-free survival in lymph-node-negative cases with estrogen-receptor-positive breast cancer (*n* = 633)

	Univariate analysis		Multivariate analysis	
	HR (95 % CI)	*P* value	HR (95 % CI)	*P* value
Age (years)		*P* = 0.0026		*P* = 0.22
≤40	1		1	
41–55	0.65 (0.46–0.91)		0.74 (0.52–1.06)	
56–70	0.55 (0.39–0.77)		0.60 (0.33–1.09)	
≥71	0.49 (0.32–0.73)		0.52 (0.27–0.99)	
Menopausal status		*P* = 0.008		*P* = 0.921
Premenopausal	1		1	
Postmenopausal	0.73 (0.58–0.92)		0.98 (0.60–1.59)	
Tumor size		*P* = 0.02		*P* = 0.045
pT1	1		1	
pT2	1.31 (1.03–1.66)		1.18 (0.92–1.51)	
pT3	1.88 (1.08–3.26)		2.07 (1.18–3.63)	
Tumor grade		*P* = 0.005		*P* = 0.015
Poor	1		1	
Unknown	0.97 (0.75–1.26)		1.05 (0.81–1.37)	
Good/moderate	0.60 (0.43–0.84)		0.66 (0.47–0.92)	
PR^a^	0.90 (0.85–0.96)	*P* = 0.001	0.94(0.88–1.00)	*P* = 0.055
median *APOBEC3B* mRNA				
High vs Low	1.55 (1.23–1.96)	*P* = 0.0002	1.32 (1.02–1.69)	*P* = 0.034

*HR* hazard ratio, *CI* confidence interval

^a^mRNA analyzed as log-transformed continuous variable

**Fig. 1:**
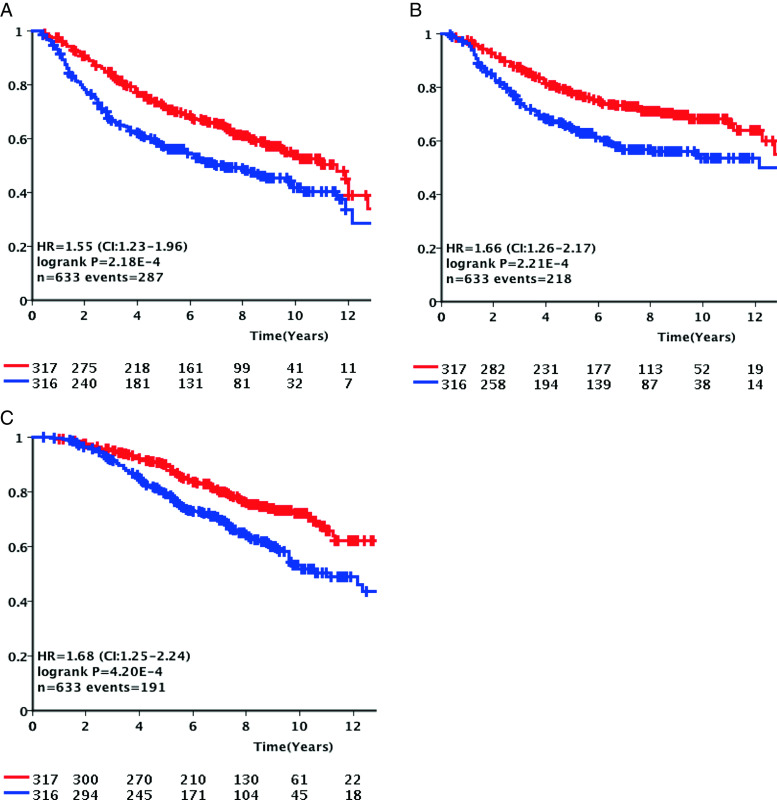
Kaplan-Meier survival analysis for the Rotterdam cohort. Kaplan-Meier curves for disease-free survival (**a**), metastasis-free survival (**b**), and overall survival analysis (**c**) for all 633 lymph-node-negative patients with estrogen-receptor-positive disease who did not receive any adjuvant systemic therapy divided at the median *APOBEC3B* mRNA expression level. *Red* and *blue* graphs represent *APOBEC3B* mRNA expression below and above the median respectively. *Y*-axis expresses cumulative survival rate (Color figure online)

Kaplan-Meier analysis (Fig. 1) was used to visualize the difference in DFS, MFS, and OS of LNN patients with ER + disease as a function of low and high *APOBEC3B* mRNA expression levels. Patients whose tumors had high *APOBEC3B* mRNA expression levels clearly fared worse than those with low expression levels (log-rank *P* value < .001 for all three analyses).

### Corroborating Data from Independent Cohorts

To confirm the results presented above in independent patient cohorts, we used public data and a sub-selection of the recently profiled prospectively collected BIG 1-98 cohort (see the “Patients and Methods” section). The validation cohorts included the METABRIC discovery and validation cohorts [8], the NKI295 cohort in which the MammaPrint prognostic signature was validated [37], and other public data profiled on Affymetrix arrays and available via http://www.kmplot.com [13]. Note that, in contrast to the retrospectively collected LNN Rotterdam discovery cohort, part of these patients received (neo)adjuvant systemic therapy and that various primary endpoints were used in the analysis.

Before analyzing the expression of *APOBEC3B* using available gene datasets, the concordance between measuring *APOBEC3B* gene expression by RT-qPCR and the gene expression on U133A microarrays was assessed (Supplementary Fig. S2). For this, we had available from our cohort a total of 309 cases with both RT-qPCR and Affymetrix gene expression data from the same specimens. Overall, these independent quantitative measurements of *APOBEC3B* expression levels correlated strongly (Spearman *r*_s_ = 0.87, *P* < .001) This reassured us that the Affymetrix probeset accurately quantifies *APOBEC3B* expression, thereby reassuring that publically available gene expression data can be used to help validate our findings.

**Fig. 2:**
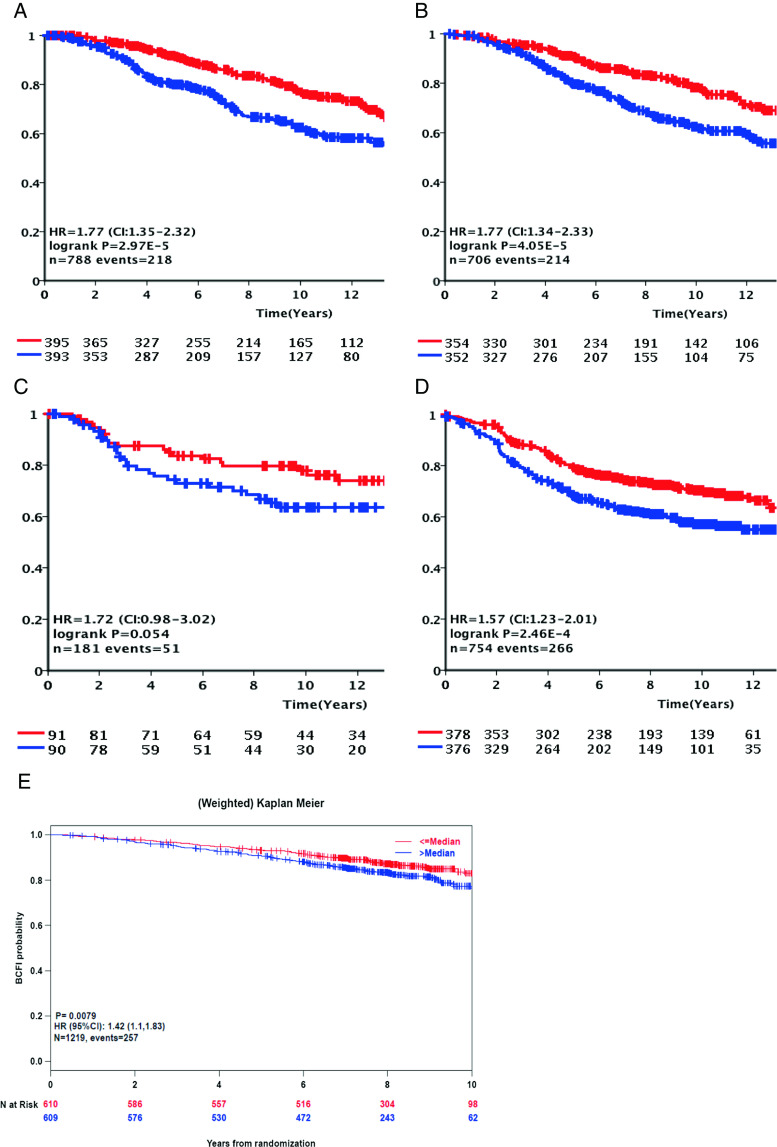
Kaplan-Meier survival analysis of validation cohorts including only cases with estrogen-receptor-positive disease. Kaplan-Meier curves for DSS in the METABRIC discovery (**a**) and METABRIC validation cohort (**b**), for DFS in the NKI cohort (**c**) and for DFS in a combined cohort including publically available Affymetrix datasets (**d**), and for BCFI in the prospective collected BIG 1-98 cohort (**e**). All cohorts were divided using the median *APOBEC3B* mRNA expression level. *Red* and *blue* graphs represent *APOBEC3B* mRNA expression below and above the median respectively. *Y*-axis expresses cumulative survival rate (Color figure online)

Retrospective analysis in five additional independent patient sets including one prospectively collected cohort of patients that received adjuvant therapy (BIG 1-98) confirmed the relation of *APOBEC3B* expression dichotomized at the median level with adverse outcome in ER + breast cancer (METABRIC discovery, 788 ER + cases OS, HR = 1.77, 95 % CI = 1.35–2.32, *P* < .0001; METABRIC validation, 706 ER + cases OS, HR = 1.77, 95 % CI = 1.34–2.33, *P* < .0001; NKI295, 181 ER + cases, HR = 1.72, 95 % CI = 0.98–3.02, *P* = .054; assembled Affymetrix microarray cohort, 754 ER + cases, DFS, HR = 1.57, 95 % CI = 1.23–2.01, *P* = .0002; and BIG 1-98, 1,219 ER + cases, BCFI, HR = 1.42, 95 % CI = 1.16–1.73, *P* = 0008). For the assembled Affymetrix microarray cohort, we further confirmed that *APOBEC3B* expression for ER − cases was not associated with outcome (122 cases, DFS, HR = 0.96, 95 % CI = 0.58–1.61, *P* = .89). Kaplan-Meier plots for the ER + cases of all five cohorts using the median *APOBEC3B* mRNA expression level as an unbiased cutoff are presented in Fig. 2.

## Discussion

The innate immune DNA cytosine deaminase APOBEC3B was recently identified as a predominant source of context dependent cytosine base substitution mutations in breast cancer [12, 33, 2, 36, 30]. This exciting finding provided a rationale for the progressive gain of passenger and potentially also driver mutations over time. This consideration lets us hypothesize that levels of *APOBEC3B* might contribute to cancer progression as mutations acquired over time would likely be *APOBEC3B* level dependent. Our results indeed show that patients with high *APOBEC3B* mRNA levels in their primary tumor more rapidly experience disease relapse. This observation was made using a retrospective cohort of patients who did not receive any systemic (neo)adjuvant treatment, which suggests that *APOBEC3B* is indeed a marker of pure prognosis and a direct contributor to breast cancer progression. However, also in patients receiving various types of adjuvant endocrine and/or chemotherapy, the analysis came out as significant, suggesting that overall, the *APOBEC3B* enzyme contributes to breast cancer progression. The prognostic value of *APOBEC3B* was especially prominent in ER + disease, suggesting that particularly in this subclass of breast cancer levels of *APOBEC3B* may contribute to cancer progression. A clear negative link between ER and *APOBEC3B* expression suggests that estrogens down-regulate this gene. However, we found no evidence for such a regulation in multiple breast cancer cell lines (Burns, Leonard, and Harris, data not shown). Why *APOBEC3B* expression would only be prognostic in ER + and not in ER − breast cancer is unclear, especially because expression is higher in ER − disease. Possibly, the mutator phenotype induced by *APOBEC3B* overexpression is not rate limiting in ER − disease, a so called C class cancer, which is often high grade, *TP53* mutant, and characterized by many copy number aberrations [5]. In line with this is our observation of a HR of 1.57 for *APOBEC3B* overexpression in luminal A breast cancer (Supplementary analysis), which is considered an M class cancer driven by mutations rather than copy number aberrations [5].

Earlier work analyzed the germline DNA of breast cancer cases versus healthy controls and reported that a deletion allele of *APOBEC3B* may be related to a higher incidence of developing breast cancer [15, 40, 18]. At first glance, this observation seems counterintuitive to the results presented here. However, incidence and progression are two clearly distinct issues. Higher incidence may be explained by the likelihood that APOBEC3B null cells are predicted to be more susceptible to viral infection and insertional mutagenesis by endogenous elements, which this protein normally serves to suppress. Our studies strongly suggest a direct role for APOBEC3B in cancer mutagenesis beyond tumor onset. Such a link with progression may also be relevant for other cancer types in which APOBEC3B is implicated in generating mutational diversity, such as bladder, lung, head and neck, and cervical cancer; however, this remains to be explored [3, 28].

Our work indicates that APOBEC3B and its associated mutator phenotype associate with poor prognosis. This result contrasts with the consequences of a different mutator phenotype in some colorectal cancers. The hereditary non-polyposis colorectal carcinoma (HNPCC) subclass of colorectal cancers typically has a germline mutation in a mismatch repair gene that results in a strong mutator phenotype (microsatellite instability) and is known to increase the incidence of cancer, but clinically results in better long-term prognosis. The main difference may be the overall level of mutation. HNPCC tumors may be more genetically “brittle” in that, while they are more likely to form, they are also unable to modulate the level of mutation once the tumor is established, making them susceptible to lethal hypermutation assisted by therapeutic intervention. HNPCC tumors cannot easily restore genetic stability because it is difficult to revert a chromosomal mutation (often germline). In contrast, *APOBEC3B*-elevated mutagenesis in breast cancer is more modest, appearing to occur gradually after the initial tumor has formed [40]. In our working model, the mutator phenotype provides cancer with a measure of sub-lethal genetic plasticity that contributes to diversifying the tumor cell population. Elevated *APOBEC3B* expression appears to be at the transcriptional level, which, depending on the tumor environment, may be up- or down-modulated. The heterogeneous population resulting from moderately elevated *APOBEC3B* expression may then yield more aggressive and drug resisting tumor cells that result in poor clinical outcomes.

Our data suggest that more aggressive treatments of ER + tumors could be considered particularly in those having high APOBEC3B. Long-lived APOBEC3B-high tumor cells, even when still dormant, will have more opportunities to accumulate mutations, evolve, escape the dormant state, outgrow, metastasize, and potentially acquire resistance during additional rounds of therapy. Stronger postoperative treatments to eradicate APOBEC3B-high, ER + cells may be effective. In addition, although still in the early stages of development [17, 21], small-molecule inhibition of APOBEC3B activity as a secondary adjuvant therapy is an attractive prospect.

## Electronic supplementary material

Below is the link to the electronic supplementary material.

ESM 1 (DOCX 293 kb)
