# Supplementary material for: Elevated APOBEC3B Correlates with Poor Outcomes for Estrogen-Receptor-Positive Breast Cancers
Source: Horm Cancer. 2014 Aug 15;5(6):405–13. doi: 10.1007/s12672-014-0196-8 (PMC4228172; doi:10.1007/s12672-014-0196-8)
Supplement: Supplementary file 1 — (DOCX 293 kb) [file 12672_2014_196_MOESM1_ESM.docx]

**Supplementary data.**

**Supplementary Diagram S1. Study design and patient subsets analyzed for *APOBEC3B* mRNA expression levels.**

**Rotterdam cohort: All fresh-frozen breast cancer cases diagnosed between 1978 and 2000 in our Rotterdam tissue bank (detailed inclusion criteria see methods section); Subselection of Lymph node breast cancer cases with ER-positive tumors (n=633). None of these pateints received adjuvant systemic therapy (standard of care at that time in the Netherlands). This cohort was evaluated with RT-qPCR for a relationship with tumor aggressiveness. The confirmation cohorts included the ER positive cases from the METABRIC study (Discovery (n=788) and Validation cohort (n=706) analyzed seperately), from the NKI study in which Mammaprint was validated (n=181), from publically available Affymetrics datasets present in kmplot (n=754) Platforms to quantify APOBEC3B are indicated which include RT-qPCR, Illumina HT-12 v4, Agilent and Affymetrics. Enpoints include: disease-free survival (DFS), metastasis-free survival (MFS), overall survival (OS), disease specific survival (DSS), Breast cancer free interval (BCFI). Type of tissue used for the studies included formalin fixed paraffin embedded material (FFPE) and freshly snap frozen tissue (FF).**

**Additional clinical info Rotterdam cohort.** Of the patients included in the study, 829 (55.6%) developed local regional and/or distant metastasis during follow-up and 697 (46.7%) distant metastases were counted as events in the disease-free survival (DFS) and metastasis-free survival (MFS) analysis, respectively. Six hundred twenty three patients (41.7%), deceased due to their breast cancer, were counted as events in the analysis for overall survival (OS). Patient who had died without evidence of disease or who were lost during follow-up were censored at last follow-up in DFS, MFS and OS analysis. We used our previously described *ESR1* and *PR* mRNA cut points[[3](#_ENREF_3)] to define tumors as steroid hormone receptor positive at 0.2 for ER and 0.1 for progesterone receptor (PR).

Details of qRT-PCR

In brief, only material from 30 µm tissue sections containing at least 30% invasive tumor cell nuclei were processed with RNA Bee (Tel Test, Thermo Fisher Scientific Inc.) to extract total RNA. After cDNA synthesis with the RevertAid H Minus First Strand cDNA Synthesis Kit from Thermo Fisher Scientific Inc, followed by an RNAse H step (Ambion, Life Technologies) to degrade the remaining RNA, qPCR reactions were performed using a Mx3000P^TM^ Real-Time PCR System (Agilent, Amsterdam, The Netherlands). PCR reactions were done in a final volume of 25 µL containing cDNA synthesized from 5 to 15 ng of total RNA, 330 nM forward and reverse primer and 12.5 µL Absolute™ QPCR SYBR® Green mastermix containing ROX (Abgene Limited, Epsom, UK). After 15 min of denaturation and activation of the Taq-DNA polymerase, PCR products were amplified in 35 cycles with 15 sec of denaturing at 95°C, 30 sec of annealing at 62°C followed by data acquisition at 72°C and 79°C. Validations performed to ensure PCR specificity were done as described [[3](#_ENREF_3)]. In brief, when amplification rounds for a specific target exceeded 35 cycles, quantities were considered to be undetectable and were set at 50% of the lowest expression level measurable at the quantification detection threshold (Cq = 0.01). Besides a negative genomic DNA control sample, a standard curve of a serially diluted cDNA of a pooled breast tumor sample was included in each PCR plate and used to control the PCR efficiency and to harmonize the data in between plates. Concentrations of the target genes, expressed relative to our reference gene set consisting of *hydroxymethylbilane synthase* (*HMBS*), *hypoxanthine phosphoribosyltransferase 1* (*HPRT1*) and *TATA-box binding protein* (*TBP*), were quantified as follows: mRNA target = 2^Cq reference gene set – Cq target gene^. All primer sequences are listed in Supplementary Table 1.

**Rotterdam Primers**

**Univ. of Michigan Primers**

**Supplementary Figure S1: Specificity of the primers to quantify *APOBEC3B* mRNA.** To determine the specificity of the RT-qPCR assay to quantify *APOBEC3B* mRNA copy DNA of 7 family members was used as input to be quantified by RT-PCR using the specific primers for *APOBEC3B* developed by Rotterdam (Erasmus MC) and used in the current study (grey bar) and the validated primers developed by Univ. of Michigan as described previously (back bar) Refsland et al. [[2](#_ENREF_2), [1](#_ENREF_1)]. The figure shows that quantification of *APOBEC3B* mRNA by RT-qPCR using both primer pairs are equally efficient and highly specific, as they do not cross react with any other human APOBEC3 family member.

***Methodology to supplementary Figure S1***

To validate the *APOBEC3B* mRNA RT-qPCR assay used in the current study, we compared it directly with a previously validated assay using cDNAs representing each of the 7 human *APOBEC3* family members as templates [[2](#_ENREF_2), [1](#_ENREF_1)]. Linearized *APOBEC3* cDNA plasmids were used to determine the primer pair efficiencies for specific quantification of *APOBEC3B*. Quantification was done using the Roche Universal Probe Library (UPL) and a Roche Lightcycler 480 instrument, as described [[2](#_ENREF_2), [1](#_ENREF_1)]. The new primer set (see below; supplementary Table 1) with an amplicon size of 111 bp has the same specificity and efficiency as the previously reported primer set with an amplicon size of 257 bp (Supplementary Figure S1).


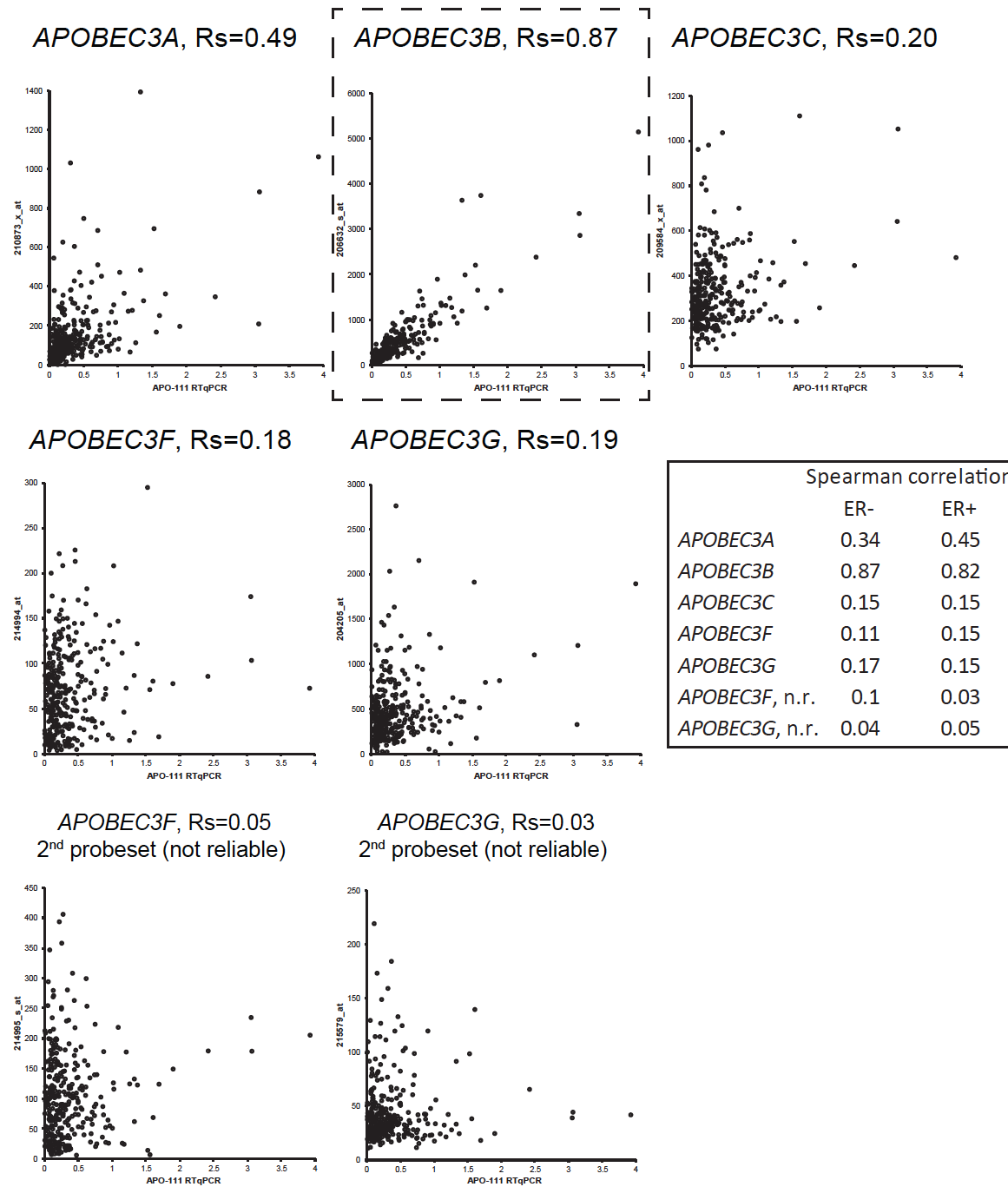


**Supplementary Figure S2. Correlation between APOBEC3B RTqPCR and microarray values for the indicated array probe sets.**

Samples from 309 breast cancers (Rotterdam) were subjected to analysis by both RT-qPCR (this study) and Affymetrix U133A chip (reported previously [[4](#_ENREF_4), [5](#_ENREF_5)]). The Spearman r_s_ value is strong for *APOBEC3B*, but not for other *APOBEC3* probes indicating agreement between RT-qPCR and Affymetrix U133A chip approaches to quantify *APOBEC3B* mRNA expression. However, the intermediate correlation for *APOBEC3A* is consistent with cross-reactivity of the *APOBEC3A* microarray probe set, as predicted previously, in which 2 *APOBEC3A* probes have 24/25 nucleotides identity and 2 others have 22/25 nucleotides identity with *APOBEC3B* [[1](#_ENREF_1)].

**Supplementory Table S1. All primer sequences used in the current study.**

| **Gene** | **F  Primer sequence** | **R  Primer sequence** |
| --- | --- | --- |
| ***APOBEC3B*** | CGCCAGACCTACTTGTGCTA | GCCACAGAGAAGATTCTTAGCC |
| ***ESR1*** | ATCCTACCAGACCCTTCAGTG | GCCAGACGAGACCAATCATC |
| ***HMBS*** | CATGTCTGGTAACGGCAATG | GTACGAGGCTTTCAATGTTG |
| ***HPRT1*** | TATTGTAATGACCAGTCAACAG | GGTCCTTTTCACCAGCAAG |
| ***PGR*** | CAAGTTAGCCAAGAAGAGTTC | ACTTCGTAGCCCTTCCAAAG |
| ***TBP*** | TTCGGAGAGTTCTGGGATTG | ACGAAGTGCAATGGTCTTTAG |

**Supplementary table S2: Univariate and multivariate analysis for metastasis-free survival in Lymph-node negative cases with estrogen receptor positive breast cancer (n=633)**

**Univariate analysis Multivariate analysis**

HR (95% CI) *P*-value HR (95% CI) *P*-value

**Age (years)** .038 .24

≤ 40 1 1

41-55 0.90 (0.60-1.35) 1.05 (0.69-1.60)

56-70 0.69 (0.46-1.05) 0.71 (0.37-1.39)

≥ 71 0.56 (0.34-0.91) 0.56 (0.27-1.16)

**Menopausal status** .034 .841

Premenopausal 1 1

Postmenopausal 0.75 (0.57-0.98) 1.06 (0.62-1.78)

Tumor size .091 .091

pT1 1 1

pT2 1.25 (0.95-1.64) 1.15 (0.87-1.53)

pT3 1.84 (0.99-3.44) 1.97 (1.04-3.71)

Tumor grade .00099 .00099

Poor 1 1

Unknown 0.98 (0.73-1.31) 1.11 (0.82-1.49)

Good/Moderate 0.50 (0.34-0.76) 0.54 (0.36-0.82)

PR**^†^** 0.90 (0.84-0.96) .002 0.93 (0.86-1.00) .065

Median *APOBEC3B* mRNA

High vs. low 1.66 (1.26-2.17) .0002 1.43 (1.07-1.91) .015

**^†^**  mRNA analyzed as log-transformed continuous variable.

**Supplementary table S3: Univariate and multivariate analysis for overall survival in Lymph-node negative cases with estrogen receptor positive breast cancer (n=633)**

**Univariate analysis Multivariate analysis**

HR (95% CI) *P*-value HR (95% CI) *P*-value

**Age (years)** .42 .20

≤ 40 1 1

41-55 0.94 (0.60-1.49) 1.04 (0.65-1.68)

56-70 0.89 (0.56-1.41) 0.68 (0.33-1.39)

≥ 71 1.24 (0.75-2.03) 1.01 (0.48-2.13)

**Menopausal status** .327 .244

Premenopausal 1 1

Postmenopausal 1.16 (0.86-1.55) 1.40 (0.79-2.47)

Tumor size .20 .24

pT1 1 1

pT2 1.16 (0.86-1.55) 1.00 (0.74-1.35)

pT3 1.83 (0.95-3.52) 1.84 (0.94-3.58)

Tumor grade .68 .23

Poor 1 1

Unknown 0.93 (0.67-1.27) 0.95 (0.69-1.32)

Good/Moderate 0.64 (0.43-0.95) 0.71 (0.47-1.07)

PR**^†^** 0.88 (0.81-0.94) .002 0.86 (0.81-0.94) .005

Median *APOBEC3B* mRNA

High vs low 1.68 (1.26-2.24) .0004 1.44 (1.06-1.96) .02

**^†^**  mRNA analyzed as log-transformed continuous variable.**Supplemental analysis: Association with molecular subtype**

Kmplot allows selection of samples according to intrinsic subtypes. Subtypes are defined as basal (ER negative/Her-2 low), luminal A (ER positive/Her-2low/MKI67low), luminal B (ER positive/Her-2low/MKI67high) and HER2+ (Her-2 high) based on the gene expression of HER2, ER, and MKI67. Using kmplot of all available cases, analysis of DFS indicates that expression of *APOBEC3B* dichotomized at the median level is not prognostic in basal breast cancer (HR=0.94, 95%CI=0.72-1.21, *P*=.61, n=581) but is, in contrast, clearly prognostic in luminal A (HR=1.57; 95%CI=1.31-1.89, *P*=1.1E-6, n=1,678) and to a lesser extent in luminal B breast cancer (HR=1.26, 95%CI=1.03-1.54, *P*=.027, n=989). In HER2+ disease differential expression of *APOBEC3B* was not significantly associated with DFS (HR=1.36, 95%CI=0.89-2.07, *P*=.15, n=207), but low numbers limit the power.

**References.**

1. Burns, M. B., L. Lackey, M. A. Carpenter, A. Rathore, A. M. Land, B. Leonard, E. W. Refsland et al. . 2013. APOBEC3B is an enzymatic source of mutation in breast cancer. *Nature* 494 (7437):366-370.

2. Refsland, E. W., M. D. Stenglein, K. Shindo, J. S. Albin, W. L. Brown, and R. S. Harris. 2010. Quantitative profiling of the full APOBEC3 mRNA repertoire in lymphocytes and tissues: implications for HIV-1 restriction. *Nucleic Acids Res* 38 (13):4274-4284.

3. Sieuwerts, A. M., P. A. Usher, M. E. Meijer-van Gelder, M. Timmermans, J. W. Martens, N. Brunner, J. G. Klijn, H. Offenberg, and J. A. Foekens. 2007. Concentrations of TIMP1 mRNA splice variants and TIMP-1 protein are differentially associated with prognosis in primary breast cancer. *Clin Chem* 53 (7):1280-1288.

4. Wang, Y., J. G. Klijn, Y. Zhang, A. M. Sieuwerts, M. P. Look, F. Yang, D. Talantov et al. . 2005. Gene-expression profiles to predict distant metastasis of lymph-node-negative primary breast cancer. *Lancet* 365 (9460):671-679.

5. Yu, J. X., A. M. Sieuwerts, Y. Zhang, J. W. Martens, M. Smid, J. G. Klijn, Y. Wang, and J. A. Foekens. 2007. Pathway analysis of gene signatures predicting metastasis of node-negative primary breast cancer. *BMC Cancer* 7 (1):182-195.
